# Single Point Insulin Sensitivity Estimator (SPISE) As a Prognostic Marker for Emerging Dysglycemia in Children with Overweight or Obesity

**DOI:** 10.3390/metabo13010100

**Published:** 2023-01-07

**Authors:** Robert Stein, Florian Koutny, Johannes Riedel, Natascha Dörr, Klara Meyer, Marco Colombo, Mandy Vogel, Christian Heinz Anderwald, Matthias Blüher, Wieland Kiess, Antje Körner, Daniel Weghuber

**Affiliations:** 1 Center for Pediatric Research, University Hospital for Children and Adolescents, Medical Faculty, University of Leipzig, 04103 Leipzig, Germany; 2 Helmholtz Institute for Metabolic, Obesity and Vascular Research (HI-MAG), University Hospital Leipzig, 04103 Leipzig, Germany; 3 Department of Pediatrics, Paracelsus Private Medical University, Muellner Hauptstrasse 48, 5020 Salzburg, Austria; 4 Department of Gastroenterology, Hepatology and Rheumatology, University Hospital St. Pölten, 3100 St. Pölten, Austria; 5 Leipzig Research Center for Civilization Diseases (LIFE Child), Medical Faculty, University of Leipzig, 04103 Leipzig, Germany; 6 Division of Endocrinology and Metabolism, Department of Internal Medicine III, Medical University of Vienna, 1090 Vienna, Austria; 7 Health Care Center Arnoldstein, 9601 Arnoldstein, Austria

**Keywords:** SPISE, childhood obesity, dysglycemia, early-onset diabetes, prediabetes, type 2 diabetes, insulin resistance

## Abstract

The single point insulin sensitivity estimator (SPISE) is a recently developed fasting index for insulin sensitivity based on triglycerides, high density lipoprotein cholesterol, and body mass index. SPISE has been validated in juveniles and adults; still, its role during childhood remains unclear. To evaluate the age- and sex-specific distribution of SPISE, its correlation with established fasting indexes and its application as a prognostic marker for future dysglycemia during childhood and adolescence were assessed. We performed linear modeling and correlation analyses on a cross-sectional cohort of 2107 children and adolescents (age 5 to 18.4 years) with overweight or obesity. Furthermore, survival analyses were conducted upon a longitudinal cohort of 591 children with overweight/obesity (1712 observations) with a maximum follow-up time of nearly 20 years, targeting prediabetes/dysglycemia as the end point. The SPISE index decreased significantly with age (−0.34 units per year, *p* < 0.001) among children and adolescents with overweight and obesity. Sex did not have an influence on SPISE. There was a modest correlation between SPISE and established fasting markers of insulin resistance (R = −0.49 for HOMA-IR, R = −0.55 for QUICKI-IR). SPISE is a better prognostic marker for future dysglycemia (hazard ratio (HR) 3.47, 95% confidence interval (CI) 1.60–7.51, *p* < 0.01) than HOMA-IR and QUICKI-IR (HR 2.44, 95% CI 1.24–4.81, *p* < 0.05). The SPISE index is a surrogate marker for insulin resistance predicting emerging dysglycemia in children with overweight or obesity, and could, therefore, be applied to pediatric cohorts that lack direct insulin assessment.

## 1. Introduction

Overweight and obesity, accompanied by metabolic syndrome and related comorbidities such as insulin resistance (IR), are a worldwide health burden adults as well as in children and adolescents [1]. Epidemiological studies have shown that up to 17.9% of children and adolescents in Europe have overweight or obesity [1]. Childhood obesity does not only lead to a high economic burden, but also to increased future morbidity and mortality [2,3].

There is ample evidence that children with obesity have a higher risk of developing insulin resistance and metabolic syndrome [4,5]. The pathogenesis of type 2 diabetes (T2D) is multifactorial; however, obesity is considered the most significant risk factor for developing IR [6]. Nevertheless, large epidemiological studies have shown that not all children with obesity have IR, and that T2D can also occur in children without obesity [7].

Therefore, the identification and screening of those at risk for future T2D is a crucial step to guide early prevention and treatment modification. Various indexes to screen for early IR and T2D have been evaluated in the pediatric population [8,9,10,11]. The gold standard for measurement of IR and insulin sensitivity (IS) is the euglycemic hyperinsulinemic clamp test [12]. However, this test is very time-consuming, expensive, and uncomfortable for the patient [13].

Thus, less time-consuming and simpler approaches, such as the quantitative insulin sensitivity check index (QUICKI), or its reciprocal, the QUICKI-IR [14], as well as the homeostatic model assessment of insulin resistance (HOMA-IR) [15], have been developed. However, these indexes require insulin measurements, which are expensive and not universally available [16].

Standards for assessing IR and IS in children and adolescents are still lacking. Thus, there is a need to evaluate new, less expensive indices with a high specificity for IR and IS in the pediatric population. Furthermore, new scores are needed for epidemiological studies in cohorts lacking insulin measurements to calculate the prevalence and incidence of T2D in the pediatric population worldwide. 

A new approach to estimate IR is the triglycerides/high-density lipoproteins cholesterol ratio (TG/HDL-C) [16]. However, studies have shown that this score has lower specificity and higher variability compared to standard methods [13,17]. Recently, Paulmichl et al. have developed a new index called the single point insulin sensitivity estimator (SPISE), redefining the TG/HDL-C ratio [18]. This index estimates IR based on single fasting samples of triglycerides (TG), high density lipoprotein cholesterol (HDL-C), and body mass index (BMI) [18]. The score was validated with the euglycemic clamp method on adults and post-pubertal adolescents [18]. SPISE showed better results than the TG/HDL-C ratio and similar results to HOMA-IR and QUICKI in this analysis among juveniles and adults [18]. However, its validity in children of younger ages has not been systematically studied yet.

Herein, we evaluated the SPISE index in a cohort of children and adolescents with overweight or obesity and, therefore, a high likelihood of insulin resistance. We addressed the question of whether SPISE is influenced by age and sex, and whether it is an appropriate surrogate marker for IR during childhood, by correlating it to established fasting IR indexes and testing its utility as a prognostic marker for emerging dysglycemia. 

## 2. Material and Methods

### 2.1. Study Desing and Study Population

Data were obtained between April 1999 and September 2022 from the Leipzig Childhood Obesity Cohort (recruited from the local obesity outpatient clinic) and the LIFE Child Cohort (recruited from the urban population around the city of Leipzig, which have been described elsewhere [19]). The studies were approved by the local ethics committee number, Leipzig Childhood Obesity Cohort: institutional review board (IRB-Nr): 007/04, NCT04491344), and the LIFE Child Cohort, IRB-Nr: 265-10-19042010; NCT02550236). 

Doublets in between cohorts were excluded. Written informed consent was provided by the legal guardians as well as the subjects themselves from the age of 12 years. All studies met the ethical standards of the Declaration of Helsinki, as revised in 2008, and have been approved by the institutional review board of the Medical Faculty of the University Leipzig, Germany. 

**Figure 1:**
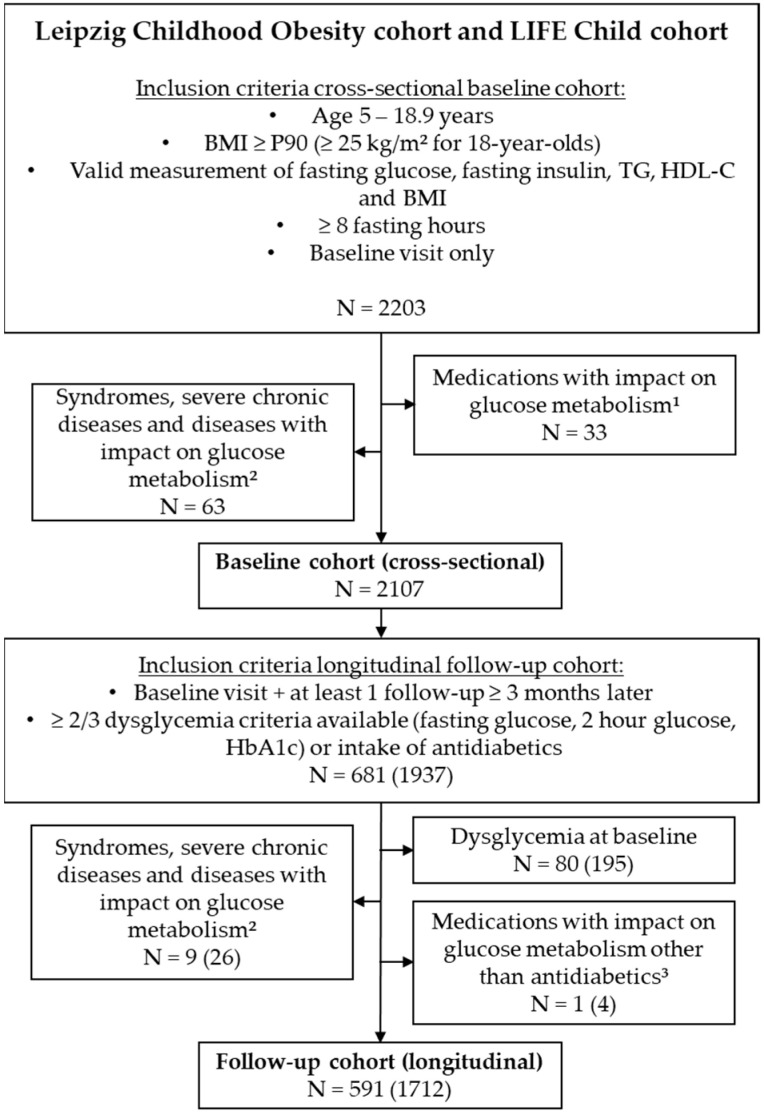
Selection of study population. ^1^ antidiabetics, growth hormone, systemic glucocorticoids; ^2^ panhypopituitarism, malignant diseases, bariatric surgery, spinal muscular atrophy, phenylketonuria, pancreatitis, strong developmental delay, cerebral paresis, acute infection, tuberoid sclerosis, pancreatitis, Crohn’s disease, Celiac disease, syndromes (Trisomy 21, Prader Willi syndrome, Bardet Biedl syndrome, Monogenic obesity, 15q13.3 microdeletion syndrome, monogenic diabetes, Beckwith Wiedemann syndrome, Kabuki syndrome, Trichorhinophalangeal syndrome, Klinefelter syndrome, Cowden syndrome, Poland syndrome, Hypochondroplasia); ^3^ growth hormone, high density lipoprotein cholesterol; 90th percentile; triglycerides.

We included 2203 children and adolescents between 5 and 18.9 years of age with overweight or obesity (defined as a body mass index SD score (BMI–SDS) ≥ 1.28 or BMI > 25, if 18 years old), a valid assessment of fasting insulin, fasting glucose, TG, HDL-C, and BMI, as well as at least 8 h of starvation prior to study participation (Figure 1). Among these, 2107 participants were free of syndromes, medications, or diseases with a potential impact on glucose metabolism, and were selected for further cross-sectional analyses (baseline cohort). 

For longitudinal survival analyses, we considered 681 subjects out of the baseline cohort who had at least one follow-up visit after more than 3 months of assessing glucose metabolism (measurement of fasting glucose, HbA1c, and/or 2 h glucose). After exclusion of syndromes and/or medications and diseases with an impact on glucose metabolism, 591 subjects with 1712 observations remained for further analyses (follow-up cohort). Notably, antidiabetic medications were not excluded for follow-up visits, as they defined the end-point dysglycemia in the survival analysis. 

### 2.2. Anthropometric and Laboratory Assessment

Height and weight were assessed by trained staff members using three repeated measurements to the nearest of 0.1 decimal units. BMI was calculated as weight [kg]/(height [m])^2^ and transformed into BMI-standard deviation scores (SDS)/percentiles using age- and sex-specific references values of the German population [20]. Pubertal status was determined according to Tanner [21] by trained staff members and then categorized into five pubertal stages, ranging from 1 (prepubertal) to 5 (complete maturity). For oral glucose tolerance testing, subjects ingested 1.75 g/kg body weight dextrose (maximum 75 g) after a 10 h overnight fast. 

All laboratory assessments were performed immediately after study participation by the certified local laboratory of the university hospital Leipzig. Insulin serum concentrations were determined by the Cobas 8000 (Roche Diagnostics, Mannheim, Germany) and LIAISON (DiaSorin, Saluggia, Italy) analyzers. Glucose was either measured in serum by a Cobas 8000 analyzer (Roche Diagnostics, Mannheim, Germany) or in hemolysates by the automated laboratory analyzer Super GL speedy using an enzymatic-amperometric method. We confirmed comparability of respective methods by Bland–Altman plots and Passing–Bablok regression (data not shown). TG and HDL-C were assessed by enzymatic color assay in a Cobas analyzer (Roche Diagnostics, Mannheim Germany). The SPISE score was calculated as follows:

SPISE = 600 × HDL-C [mg/dL]^0.185^/TG [mg/dL]^0.2^/BMI [kg/m^2^]^1.338^ (1)

HOMA-IR and QUICKI-IR served as established fasting surrogates for insulin resistance.

HOMA-IR = fasting glucose [mmol/L] × fasting insulin [mU/L]/22.5(2)

QUICKI-IR = log (fasting glucose [mmol/L]) × log (fasting insulin [mU/L])(3)

where log is the natural logarithm.

### 2.3. Statistical Analysis

All analyses were performed with the R statistical package, version 3.5.0. Graphical evaluation of SPISE scores over age was facilitated by local polynomial regression fitting, using two degrees of polynomials and including 60% of the neighbouring data points, weighted according to their distance in a tricubic manner. 

For cohort characteristics, continuous parameters were described by mean and standard deviation (SD). The association of age and sex with SPISE was tested by linear regression analysis, whereas SPISE was considered as the dependent variable and age and sex as the independent variables. For correlation analysis between SPISE and other markers of insulin resistance, Pearson product–moment correlation was used. A *p*-value of <0.05 was considered statistically significant.

Survival analyses were conducted with the R package. The risk for emerging dysglycemia was assessed by Kaplan–Meier analyses and cox proportional hazard regression. The stratification of SPISE score into the lowest and the highest quartile had to take the age-dependency of the SPISE score into account. Therefore, we divided the cohort into three age groups (5–8.9 years, 9–13.9 years, 14–18.9 years) and quartiles were calculated for each age group separately for SPISE as well as for HOMA-IR and QUICKI-IR. The endpoint dysglycemia was defined as intake of antidiabetics or meeting at least two out of three prediabetes criteria according to current guidelines from the American Diabetes Association [22]: fasting glucose ≥ 5.6 mmol/L, 2 h glucose ≥ 7.8 mmol/L, or HbA1c ≥ 5.7%. We considered prediabetes criteria rather than diabetes criteria as the end point for two reasons: (i) overt diabetes is still uncommon during childhood (only 2.5% of cases, Table A1), (ii) antidiabetic medications such as Metformin had often already been prescribed for prediabetic conditions in clinical practice; thus, it was difficult to distinguish whether the intake of antidiabetic medications was due to prediabetes or diabetes. 

## 3. Results

### 3.1. Baseline Characteristics

We aimed to evaluate the SPISE index in children and adolescents at risk for insulin resistance and (pre)diabetes. Therefore, we selected 2107 patients with overweight or obesity from the Leipzig Childhood Obesity and LIFE Child cohort who were free of medications or underlying diseases with an impact on glucose metabolism (other than obesity). Characteristics of those subjects at baseline are summarized in Table 1. Ages ranged from 5 to 18.44 years, and sex was equally spread among the cohort (49.5% male subjects). 

### 3.2. SPISE Index Is Dependent of Age, but Not of Sex during Childhood

**Figure 2:**
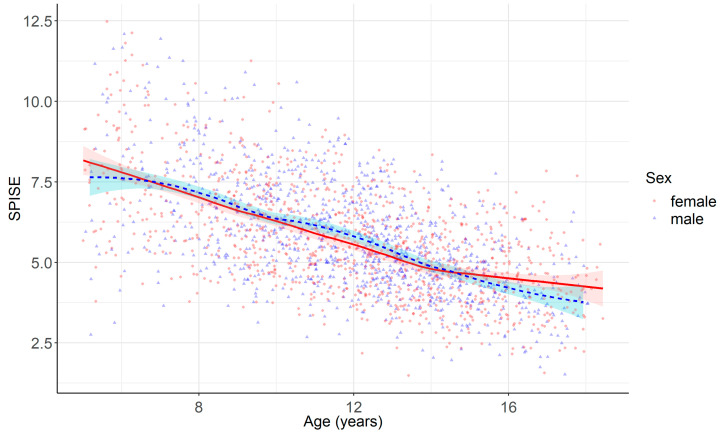
Distribution of SPISE over age. Curved lines and ribbons represent local polynomial regression fitting with 95% confidence intervals.

SPISE decreased continuously with age (−0.34 units per year) among children and adolescents with overweight or obesity (Figure 2, Table 2). When comparing age-dependent dynamics between SPISE and its components (BMI, HDL-C, TG) separately, this age dependency mainly mirrors the increase in absolute BMI and, to a smaller extend, the increase in TG during childhood and adolescence (data not shown). In contrast, no sex differences were observed (Figure 2, Table 2). Furthermore, and in contrast with established fasting indexes such as HOMA-IR and QUICKI-IR, there was no peak in SPISE during puberty (Figure 2, data for each pubertal stage are not shown). 

### 3.3. Cross-Sectional Association of SPISE Score with Established Markers of Insulin Resistance

We compared SPISE with established fasting markers of insulin resistance by Pearson product–moment correlation (Table 3). There was a moderate and significant negative association between the insulin sensitivity marker SPISE and the insulin resistance markers HOMA-IR (R = −0.49) and QUICKI-IR (R = −0.55). In comparison, HOMA-IR and QUICKI-IR correlated more strongly with each other (R = 0.88), which is not surprising, as they used the same variables (fasting glucose and fasting insulin). 

### 3.4. Longitudinal Prediction of Dysglycemia

**Figure 3:**
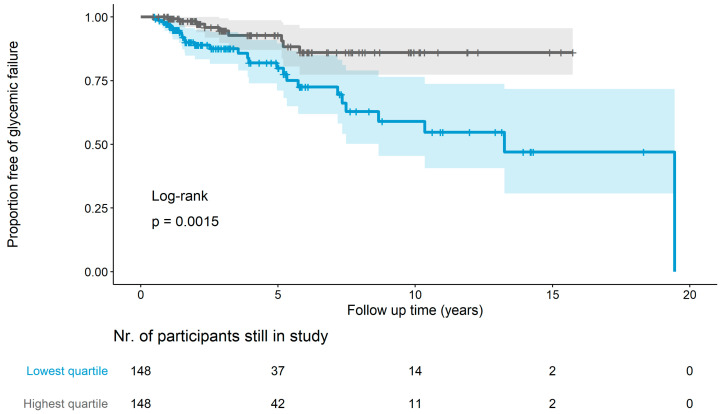
Longitudinal prediction of dysglycemia by SPISE. Ribbons around the survival curves represent the 95% confidence interval, and ticks represent right-censored data.

As insulin resistance is a crucial step preceding the development of T2D, we tested the utility of SPISE as a prognostic marker for future dysglycemia. Therefore, we selected 591 subjects derived from the baseline cohort who underwent assessment of glucose metabolism during at least one follow-up visit. The maximum follow-up time was 19.55 years, ranging up to age of 35 years (mean follow-up time 3.68 years, median 2.18 years). The characteristics of this longitudinal follow-up cohort are summarized in Table 1. During follow-up, 79 out of 591 subjects (13.37%) had an event of glycemic failure (Table A1). Participants with a low SPISE at baseline (lowest quartile) were three times as likely to develop dysglycemia during follow-up as participants with the highest quartile of SPISE, even after adjustment for age and sex (HR 3.47 (95% CI 1.60–7.51), *p* < 0.01, Figure 3, Table 4). This relationship also remained when using SPISE as a continuous variable rather than a categorial variable (HR 0.77 (95% CI 0.66–0.9), *p* < 0.01, Table 4). Furthermore, SPISE was a stronger predictor for future dysglycemia than established markers of insulin resistance, such as HOMA-IR and QUICKI-IR (HR 2.44 (95% CI 1.24–4.81), *p* < 0.05). Notably, HRs when comparing the highest vs. the lowest quartile of HOMA-IR and QUICKI-IR were the same as the values of both indexes ranked in the same order, despite having different absolute values.

## 4. Discussion

We were able to show that SPISE is dependent on age, but not on sex, and correlates moderately with established fasting markers of insulin resistance HOMA-IR and QUICKI-IR among children and adolescents with overweight and obesity. Importantly, we find that SPISE is a better predictor for emerging dysglycemia than established markers of insulin resistance among children and adolescents with overweight or obesity. 

The SPISE index has been evaluated by previous studies [23,24]. A recent study by Cederholm et al. demonstrated a significant correlation with the clamp method in a cohort of Swedish adults (*n* = 1049, age at baseline 71 years) [24]. SPISE has been shown to significantly predict future coronary heart disease, similarly to HOMA-IR and QUICKI-IR [24]. Furthermore, a lower SPISE index was significantly associated with T2D, abdominal obesity, and higher levels of adiponectin and C-reactive protein in adults and adolescents [24,25]. Furthner et al. have shown that SPISE is a surrogate marker for non-alcoholic fatty liver disease in pubertal adolescents aged 10 to 18 years with a Tanner stage of 2 to 4 [26]. SPISE performed better than HOMA-IR when compared to liver MRI as a gold standard [26]. Barchetta recently examined the relationship between the SPISE index and various insulin sensitivity indicators in children with overweight/obesity [23]. The current study, with more than twice as many participants in the cross-sectional analysis and three times as many subjects in the follow-up analysis, was able to confirm the previously presented results of the study mentioned above [23]. The results of both studies show that a low SPISE index reliably predicts the development of impaired glucose regulation in children with obesity [23]. In the present study, the endpoint “impaired glucose tolerance” was defined even more precisely, with 2 out of 3 prediabetes criteria (fasting glucose, 2 h glucose, HbA1c) at follow up at least 3 months later. Barchetta et al. defined only an isolated disturbed fasting glucose, insulin value, or 2 h glucose value as impaired glucose regulation, and the intake of metformin was not taken into account [23]. 

Despite the validation described above, data from larger epidemiological studies in the pediatric population were still lacking. Therefore, the current study, with 2107 participants, can make a significant contribution to the evaluation of the SPISE index in the pediatric population. The range of SPISE for late adolescents 16 to 18 years old was comparable to results reported by Paulmichl et al. [18]. Nevertheless, results of this study have shown that the SPISE index has a strong age dependency during childhood. Data are in line with the results reported by Barchetta et al. evaluating the performance of SPISE in a population of children from Italy with and without obesity [23]. The SPISE index was significantly correlated to age with a correlation coefficient of –0.57 (*p* < 0.001) in this cohort [23]. Based on the results of the current study, the age dependency of SPISE mainly mirrors the age-dependent increase in absolute BMI during childhood. Therefore, future studies should evaluate age-adjusted reference ranges for the SPISE index. Besides age-adjusted references, the evaluation of a new score using BMI-SDS instead of BMI in the pediatric population could also be addressed in future studies in order to repeal a certain insecurity in the performance of the SPISE Index in different age groups as a score. Using an age- and gender-specific BMI median (BMI-SDS) allows for even more precise evaluation in the reference group. 

There is ample evidence that insulin resistance peaks during puberty [27]. However, the data of the current study showed no correlation of SPISE with different Tanner stages. Still, all three parameters contained within the SPISE index (BMI, TG, HDL-C) have previously been identified to predict insulin resistance [28,29,30]. Studies have shown that BMI, along with adipose tissue mass and whole-body insulin sensitivity, further enhances the sensitivity of the SPISE index [30]. Furthermore, it has been shown that increased visceral fat accumulation and adipose tissue dysfunction are associated with IR [30]. Therefore, the addition of BMI further enhances the sensitivity of the SPISE index, as studies have shown that the lipid and lipoprotein profiles changed in patients with insulin resistance measured by euglycemic clamps [29]. Recently, Ma et al. showed that TG levels are correlated with both insulin resistance and beta cell function in individuals with dyslipidemia alone [29]. Another study identified a TG:HDL-C ratio greater than or equal to 1.36 as an early and sensitive predictor of insulin resistance in children [28]. Therefore, integrating TG and HDL-C into the SPISE index appears attractive. 

Most importantly, SPISE index was a significant predictor of future dysglycemia in children and adolescents during 20 years of follow up. This result is consistent with the findings of Sagesaka et al. based on over 27,000 adults, showing that a low SPISE index at baseline significantly predicts the incidence of dysglycemia in adults for up to 10 years prospectively [31]. Barchetta et al. also performed longitudinal analyses, showing similar results for a 6.5-year follow-up period [23]. The study reported an independent prediction of the development of impaired glucose metabolism in the future, regardless of confounders such as sex and age, in a pediatric population [23]. Furthermore, they showed that all other insulin-derived indices of IR and IS failed to predict future incidences of dysglycemia in children and adolescents [23]. The current study has shown that the SPISE index performed equally to or even better than established markers of insulin resistance such as the QUICKI-IR and HOMA-IR. This result is consistent with previous studies in adults showing that the accuracy of the SPISE index is comparable to QUICKI-IR and HOMA-IR [18]. 

The results of the current study support the clinical significance of the SPISE index, as it reliably predicts metabolic abnormalities and emerging dysglycemia in children and adolescents. Furthermore, as mentioned above, the SPISE index performed equally to or even better than established markers such as QUICKI-IR (SPISE: HR 3.47, *p* < 0.01; QUICKI-IR: HR 2.44, *p* < 0.05). It is cost-effective and easy to collect with widely available anthropometric and laboratory values [18]. The euglycemic hyperinsulinemic clamp, representing the gold standard for measuring insulin sensitivity, is more invasive, expensive, and too difficult to perform in clinical practice [32]. Other methods to measure insulin resistance, such as the Matsuda index or the oral glucose tolerance test, are more time-consuming [33]. On the one hand, this makes the SPISE index particularly attractive for countries with limited healthcare options and, on the other hand, this Index is of great importance in the evaluation of large epidemiological cohorts with missing data on glucose metabolism. 

There were certain limitations to our study which need to be acknowledged. Given the strong age dependency of both the SPISE index and established fasting markers of insulin resistance, the most accurate approach for stratification into “high” and “low” would require the application of age-adjusted percentiles. To address this limitation, we stratified baseline indexes within three different age intervals separately. Furthermore, the performance of the SPISE index was not directly compared to the euglycemic hyperinsulinemic clamp test, which is the gold standard test for insulin resistance. 

On the other hand, a strength of this study was its large number of participants. Furthermore, to our knowledge, it is the largest study in the pediatric population presenting longitudinal follow-up data on this matter.

## 5. Conclusions

SPISE is a potential surrogate marker for insulin resistance predicting emerging dysglycemia in children with overweight or obesity, and could, therefore, be applied in pediatric cohorts that lack direct insulin assessment. Given the strong age dependency of SPISE during childhood, further research is required to establish age-adjusted reference values and/or to establish an adapted score that uses BMI-SDS rather than absolute BMI in children. 

## Figures and Tables

**Table 1:** Clinical characteristics of cross-sectional baseline cohort and longitudinal follow-up cohort at baseline.

Clinical Characteristics	Baseline Cohort		Follow-Up Cohort at Baseline	
	*N*	Value	*N*	Value
*N* male (%)	2107	1043 (49.5)	591	274 (46.36)
Age range, years	2107	5.01–18.44	591	5.01–17.56
Mean age (SD), years	2107	11.91 (±3.05)	591	11.47 (±2.83)
Mean BMI (SD), kg/m^2^	2107	29.41 (±5.84)	591	28.6 (±5.38)
Mean BMI SDS (SD)	2107	2.48 (±0.57)	591	2.43 (±0.56)
Pubertal stage,%	2107	100	591	100
1	603	28.62	191	32.32
2	416	19.72	130	22.0
3	238	11.30	65	11.0
4	213	10.10	58	9.81
5	514	24.39	124	20.98
N/A	123	5.84	23	3.89
Mean fasting glucose (SD), mmol/L	2107	5.21 (±0.58)	591	5.16 (±0.44)
Mean 2 h glucose (SD), mmol/L	1896	6.64 (±1.27)	515	6.6 (±1.14)
Mean HbA1c (SD),%	1868	5.31 (±1.22)	515	5.23 (±0.33)
Mean TG (SD), mmol/L	2107	1.21 (±0.67)	591	1.18 (±0.64)
Mean HDL-C (SD), mmol/L	2107	1.22 (±0.28)	591	1.25 (±0.29)
Mean fasting insulin (SD), pmol/L	2107	121.6 (±81.94)	591	110.95 (±68.77)
Mean SPISE (SD)	2107	5.71 (±1.73)	591	5.94 (±1.71)
Mean HOMA-IR (SD)	2107	4.13 (±3.02)	591	3.69 (±2.36)
Mean QUICKI-IR (SD)	2107	4.3 (±0.68)	591	4.22 (±0.63)

High density lipoprotein cholesterol; homeostasis model assessment of insulin resistance; quantitative insulin sensitivity check index—insulin resistance; standard deviation; single point insulin sensitivity estimator; triglycerides.

**Table 2:** Linear regression analysis with SPISE as dependent variable and sex and age as independent variables at baseline.

	Univariate Regression	Multiple Regression *
Age β-slope (per year)	−0.34	−0.34
95% CI	−0.36–(−0.32)	−0.36–(−0.32)
*p* value	<0.001	<0.001
Sex Δ β-slope (for male)	0.07	0.04
95% CI	−0.07–0.22	−0.08–0.16
*p* value	0.33	0.48

* For multiple linear regression, sex and age were both included as independent variables in one model.

**Table 3:** Pearson product–moment correlation of SPISE and established insulin indexes at baseline.

Insulin Index	SPISE	HOMA-IR
HOMA-IR	−0.49 ***	1 ***
QUICKI-IR	−0.55 ***	0.88 ***

*** *p* < 0.001.

**Table 4:** Longitudinal prediction of dysglycemia by SPISE score and established indexes of insulin resistance (Cox proportional hazard regression and log-rank test).

Insulin Index	SPISE	HOMA-IR	QUICKI-IR
*p*-value, Log-rank lowest vs. highest quartile ^A^, *N* = 296 ^B^	0.0015	0.01	0.01
HR lowest vs. highest quartile ^A^ (95% CI), *N* = 296 ^B^ Univariate Adjusted for age and sex			
	3.19 (1.5–6.81) **	2.35 (1.2–4.59) *	2.35 (1.2–4.59) *
	3.47 (1.60–7.51) **	2.44 (1.24–4.81) *	2.44 (1.24–4.81) *
HR continuous (95% CI), *N* = 591 ^C^ Univariate Adjusted for age and sex	0.84 (0.73–0.96) ** 0.77 (0.66–0.9) **	1.13 (1.04–1.24) ** 1.16 (1.05–1.27) **	1.66 (1.14–2.41) ** 1.85 (1.23–2.77) **

^A^ For HOMA-IR and QUICKI-IR, the highest quartile was compared to the lowest quartile for better direct comparison with the HR of SPISE; ^B^ For Matsuda index N = 232 and for reduced Matsuda index N = 250; ^C^ For Matsuda index N = 463 and for reduced Matsuda index N = 499; confidence interval; hazard ratio; * *p* < 0.05, ** *p* < 0.01.

## Data Availability

The datasets generated during and/or analyzed during the current study are available from the corresponding author upon reasonable request. The data are not publicly available due to restrictions, which apply to the availability of these data, which were used under license for this study. Data are available with permission from LIFE Child, Leipzig and the Leipzig Childhood Obesity Consortium.

## Appendix A

**Table A1:** Subgroups of dysglycemia.

Outcome	N (%)
Dysglycemia (total) Intake of antidiabetics ≥2/3 Prediabetes criteria positive * ≥2/3 Diabetes criteria positive **	79 (100%)
	50 (63.29%) 29 (36.71%) 2 (2.53%)

* Fasting glucose ≥ 5.6 mmol/L, 2 h glucose ≥ 7.8 mmol/L, HbA1c ≥ 5.7%; ** fasting glucose ≥ 7.0 mmol/L, 2 h glucose ≥ 11.1 mmol/L, HbA1c ≥ 6.5%.
